# Perspectives on Lyme disease among persons of color in the United States: Qualitative research findings

**DOI:** 10.1016/j.qrmh.2026.100047

**Published:** 2026-07-10

**Authors:** Stephanie A. Duench, L. Hannah Gould, Alexandra Kissling, Kathleen Beusterien, Lewis Kopenhafer, Gabriela Burgos

**Affiliations:** a US Medical Affairs, US Commercial Division, Pfizer, Collegeville, PA, USA; b Global Vaccines and Antivirals Medical Affairs, US Commercial Division, Pfizer, New York, NY, USA; c Oracle Life Sciences, Austin, TX, USA

**Keywords:** Lyme disease, Health disparities, Discrimination, Patient experiences

## Abstract

**Objective(s):**

Lyme disease is the most common vector-borne illness in the United States, with an estimated 476,000 cases diagnosed and treated annually. Although incidence is highest among White individuals, people of color are more likely to experience severe or disseminated manifestations. This study explored factors contributing to this disparity by examining experiences of people of color diagnosed with Lyme disease.

**Setting:**

Participants resided in high-incidence U.S. states.

**Participants:**

Self-identified people of color diagnosed with Lyme disease within the past 12 months.

**Methods:**

Fifteen participants completed in-depth interviews analyzed using inductive and deductive qualitative content analysis. Follow-up focus groups supported consensus-building and thematic refinement.

**Results:**

Key themes included limited awareness that people of color are at risk for Lyme disease, perceived discrimination in healthcare encounters, diagnostic delays attributed to the characteristic erythema migrans (EM) rash being less visible on darker skin, and insufficient physician knowledge regarding diverse clinical presentations. Participants also expressed a preference for racially concordant providers. Lyme disease affected physical health, emotional well-being, social relationships, and engagement in outdoor activities.

**Conclusions:**

People of color may face distinct challenges in Lyme disease recognition and management, contributing to disparities in outcomes. Findings underscore the need for targeted education for at-risk populations, patients, and providers and for equitable prevention and diagnostic strategies.

## Introduction

Lyme disease is the most common vector-borne illness in the U.S. Lyme disease is caused by the bacterium *Borrelia burgdorferi* and transmitted through the bite of an infected *Ixodes spp*. tick (Kugeler et al., 2021, Steere et al., 2016). Most cases of Lyme disease are reported in 15 high-incidence (>10 cases/100,000 persons) states and the District of Columbia in the Northeast, upper Midwest, and mid-Atlantic regions (Kugeler et al., 2024). Risk factors for Lyme disease include activities and occupations in wooded and grassy areas where ticks and their hosts reside (Gould, Fee, et al., 2024). Lyme disease can manifest with a wide range of symptoms impacting multiple organ systems including rash, fever, facial paralysis, and arthritis (Centers for Disease Control and Prevention, 2024b).

While most cases of Lyme disease are reported among White people, more severe manifestations of the disease are disproportionately found among people of color (Gould et al., 2024, Starke et al., 2023). For example, people of color are more likely than their White counterparts to be diagnosed with neurologic symptoms (Ly, 2022, Schwartz, 2017), carditis (Schwartz, 2017), and arthritis (Hunt et al., 2023, Nelson et al., 2016, Schwartz, 2017). Studies have also found that people of color are diagnosed later in the season and in settings outside of primary care, resulting in delayed treatment and increased cost of care (Gould et al., 2024, Yu et al., 2026).

These disparities are likely attributable to many factors. Racial biases and inequities in health care, particularly implicit and explicit biases (Hall et al., 2015) and differential access to treatment within the healthcare system (Artiga et al., 2023) have well-documented impacts on the experiences of people of color. Biological factors also interact with social factors, for example, the characteristic rash associated with Lyme disease is likely more challenging for healthcare providers to recognize on darker skin tones (Dodd et al., 2023, Ebede and Papier, 2006, Nolen, 2020). In addition, there may be differences in disease risk and exposure between White people and people of color based on where they live and activities in which they participate. For example, there is a higher incidence of Lyme disease in socioeconomically advantaged areas with disproportionately more White people (Greene et al., 2015, Springer and Johnson, 2018). Further, some evidence suggests that White people are more familiar with Lyme disease symptoms (Hu et al., 2019) and are more likely to know someone else with Lyme disease (Gupta et al., 2018) compared to people of color.

As research is lacking on the experiences of people of color with Lyme disease, this study sought to provide further context into this disparity. Specific objectives included a better understanding of perspectives on Lyme disease among people of color including Lyme disease awareness, details on their healthcare journeys with Lyme disease, and the impact of Lyme disease on their lives. These results could help inform prevention and education efforts on Lyme disease.

## Methods

This study involved conducting one-on-one, camera-off virtual interviews between November 2023 and February 2024 with people of color in the U.S. who had Lyme disease in the past 12 months. Following the interviews, on-camera focus groups were conducted with a subset of respondents to help provide more context around key findings. In-depth interviews were selected for their ability to reveal how participants understand and interpret (i.e., make meaning of) their experiences as people of color with Lyme disease and their interactions with their healthcare systems. In-depth interviews were also selected for their ability to elicit the emotional dimensions of social experiences that are not often evident in behavior (Lamont & Swidler, 2014).

Focus groups were used for two reasons. First, they were used to establish concordance in themes reported during the interviews. Second, because racial health disparities are often tied to sensitive issues like discrimination, mistrust in healthcare, and systemic inequities, a group setting can create a safe space where participants feel comfortable sharing their perspectives, especially when facilitated by culturally competent moderators (Ruff et al., 2005, Salisu et al., 2023). The study protocol was reviewed for ethical consideration and granted exempt status by Pearl IRB, an independent review board, in November 2023.

Eligible participants were adults (18 years or older) as well as adult caregivers of children (age 17 or younger) who could report on the experience of their child and their own experience navigating the healthcare system for their child’s care. Additional eligibility criteria included residence in one of 16 high-Lyme disease incidence jurisdictions (Connecticut, Delaware, Maine, Maryland, Massachusetts, Minnesota, New Hampshire, New Jersey, New York, Pennsylvania, Rhode Island, Vermont, Virginia, Washington, D.C., West Virginia, and Wisconsin), ability to speak English, and self-identification as a person of color (including non-White racial or ethnic identities such as Black/African American, Hispanic/Latino, Asian/Pacific Islander, and Middle Eastern/North African). In addition to self-reported race/ethnicity, participants were asked to provide a video or image to confirm their/their child’s skin color to match them with a racially/ethically concordant moderator, if possible. All participants self-reported that they or their child received a Lyme disease diagnosis in the past 12 months. In addition to reporting a diagnosis of Lyme disease, participants were asked to report their or their child’s disease manifestations (e.g., erythema migrans [EM], Lyme arthritis, neurological conditions, carditis) and the diagnostic tests used to confirm their infection (e.g., two-tiered serology blood test, synovial fluid test, cerebrospinal fluid test).

Participants were identified and recruited via social media, online forums, recruiter panels, and participant referrals. However, given the small population of people of color with Lyme disease, approaching participants via social media was more fruitful. Social media outreach targeted support groups for individuals with Lyme disease, including groups specifically created for people of color with Lyme disease. Participants were also asked to refer others who may have been interested in participation. Participants completed a screening questionnaire to determine whether they qualified for the study. Qualified participants who agreed to participate provided informed consent.

One-on-one interviews were conducted by two trained moderators on the research team who identified as a Black woman (Moderator #1) and a Latina woman (Moderator #2), respectively. Guided by a semi-structured interview protocol with a series of open-ended questions, the interviews explored participants' experiences with Lyme disease, including their awareness of the condition, initial symptoms, pathways to diagnosis, treatment journeys, and the broader impacts on their health and daily lives. To aid participants in describing their journeys, participants were shown a Blob Tree, i.e., a visual aid designed to elicit the description of emotions and experiences (Wilson & Long, 2018). The interview guide also included techniques stemming from the Psycho-Onco Emotional Anxiety (POEM) framework to help participants establish a narrative of their experience with Lyme disease (Kim et al., 2024). Specific prompts included “Imagine there is a timeline of your experience from the past to the present time. You are walking in the direction toward the past, to the first time that you were aware of your Lyme disease. Now tell me about that time,” “What was your experience with the health care system around Lyme disease?” and “What have been the most challenging aspects of your experience?” The interviews lasted approximately 60 minutes.

To facilitate participants’ comfort and willingness to share in a setting discussing their experiences, the focus group was conducted by Moderator #1. The focus group comprised five participants who had completed interviews and were selected to represent different aspects of the Lyme disease experience, such as those who had simple diagnosis experiences and had positive relationships with their healthcare providers (HCPs) as well as those who had more complex diagnosis experiences and more challenging relationships with their HCPs. This was done to ensure that a wide range of concepts could be discussed during the focus group. During the focus group, participants were presented with concepts identified in the interviews and asked to explain how these concepts fit with their lived experiences. The focus group lasted approximately two hours.

As researchers investigating racial health disparities regarding Lyme disease, the study team acknowledges the importance of critically reflecting on their positionality and its potential influence on this study. The research team comprised of mostly White authors, with one Latina author. Their professional backgrounds included a pharmaceutical company developing a Lyme disease vaccine candidate and an independent research company. Affiliation with the pharmaceutical industry may have shaped their perspectives on the role of biomedical interventions in addressing health disparities. Professional investments in Lyme disease treatment and prevention could also influence how the data have been interpreted. To mitigate against this, the study team engaged in ongoing critical discussions about their biases and prioritized participant narratives as central to its findings. Additionally, as a majority-White research team studying racial disparities, the study team acknowledges the limitations of their lived experiences in fully capturing the nuanced realities of marginalized communities. To foster an environment conducive to sharing the experience of being a person of color, moderators who identified as people of color facilitate interviews and the focus group, as explained above.

The analysis, including the development of codes and categories, was collaborative and reflective for the purpose of identifying key experiences of people of color with Lyme disease. Verbatim transcripts were provided for the one-on-one interviews and focus group. Interview data were analyzed to reveal emergent concepts before moving to consensus building in the focus group stage. The analysis was rooted in qualitative content analysis, including a deductive approach to provide answers to specific research questions and an inductive approach to explore meanings (Roller, 2019).

Qualitative data analysis software—MaxQDA (VERBI Software, 2024)—was used to store the verbatim transcripts. To begin, a member of the study team systematically read transcripts and established a coding system using MaxQDA to facilitate coding. Next, the study team periodically reviewed these codes to establish patterns, identify new concepts, and make refinements. Key concepts were grouped into categories. The preliminary round of coding and review established categories based on the discussion guide, such as “awareness” and “interactions with the healthcare system.” Key concepts, such as lack of awareness of other people of color with Lyme, experiences of White celebrities and friends, and the impact of having a doctor who is also a person of color, were grouped within these categories. While a single coder developed the initial coding system, to strengthen the reliability of the findings, the research team reviewed these codes together to discuss patterns, identify new codes, and make refinements (Beher et al., 2025). Saturation was assessed to identify code saturation in core categories (Hennink & Kaiser, 2022). Research shows that a sample size of between nine and 17 interviews is sufficient to identify the range of codes and capture relevant categories (Hennink et al., 2017, Hennink and Kaiser, 2022). By the last interview, no new concepts associated with awareness, healthcare interactions, or life impacts of Lyme disease were identified.

To situate the findings within broader social structures, ecosocial theory guided the analytic interpretation of the data. Ecosocial theory is a multilevel framework that integrates social, environmental, and biological processes to explain population health patterns (Krieger, 2012). This framework includes four key concepts: embodiment, pathways to embodiment, cumulative exposures, and agency and accountability. “Embodiment” describes the direct impact of external factors on the body—how the biological and social world becomes literally incorporated into the body. “Pathways to embodiment” describe those external factors, such as stressors or supports, through which social and environmental conditions influence patient health experiences. “Cumulative exposures” capture how experiences accumulate and interact over the life course. Finally, “agency and accountability” identify the individuals, institutions, and systems that are responsible for producing or mitigating health inequities. While four concepts exist within the ecosocial framework, three are used here due to data limitations. The current study does not engage “pathways to embodiment,” as participants’ narratives did not provide sufficient detail on the multilevel structural and biological mechanisms through which exposures were internalized over time.

Fifteen participants were interviewed, including thirteen adults and two caregivers of children under age 18. Participants were interviewed an average of eight months after their or their child’s diagnosis (range: 4–11 months).

The focus group included five of the same participants who participated in the one-on-one interviews. Table 1 reports demographic characteristics of the participants. The participants were an average of 37 years old, and the children of caregiver participants were 15 and 17 years old, respectively. Thirteen of the participants identified as Black, and nearly all resided in the Northeastern U.S. (14). Less than half of the participants (6) reported early manifestations of Lyme disease, including EM rash (3) and flu-like symptoms (3). The remainder (9) reported disseminated manifestations of Lyme disease, including arthritis (4), carditis (4), and nervous system manifestations (3).

**Table 1:** Sample Characteristics.

	**Participants (n = 15)**
Age of participant in years, mean (range) Age of child, min-max	37 (22−59) 15–17
Self-reported race, n	
Black	13
East Asian	1
Biracial	1
State of residence, n	
New York	7
Massachusetts	3
Pennsylvania	4
Minnesota	1
Type of Lyme Disease, n*	
Early manifestations	6
Erythema migrans (EM) skin rash or “Bullseye” rash	3
Flu-like Lyme disease	3
Later manifestations	9
Lyme arthritis	4
Lyme carditis	4
Neurologic Lyme disease	3
Months since diagnosis, mean (range)	8 (4−11)
Location Type, n	
A large city, population > 500,000	11
A suburb near a large city, population between 100,000 and 500,000	2
Missing	2
Patients with on-going symptoms of Lyme disease, n	5
Note: Types of Lyme disease are not mutually exclusive categories and can total more than 100%.	

## Results

**Fig. 1:**
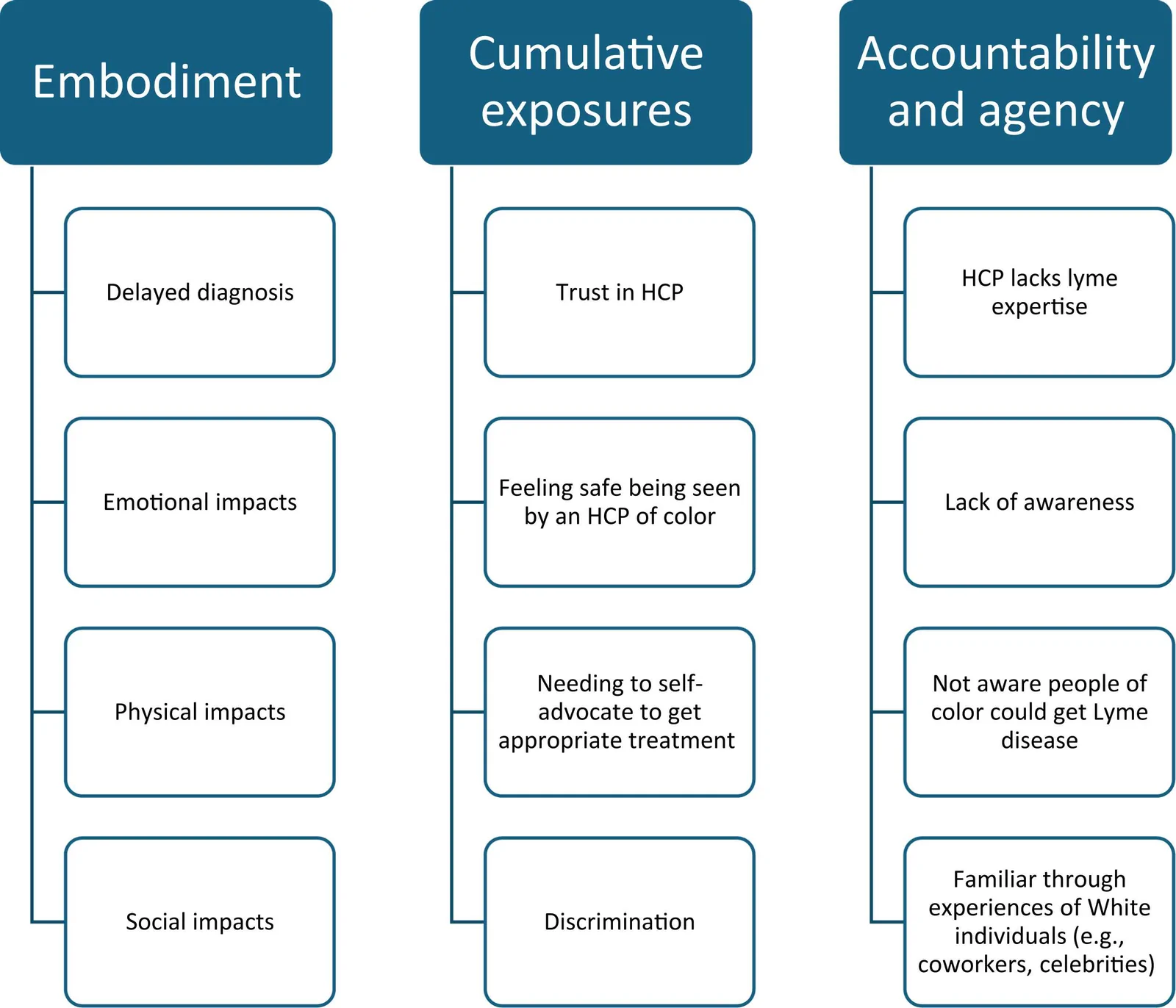
Conceptual model.

Key concepts emerging from this research were organized around the domains of the ecosocial theory framework and are presented below (Fig. 1):

## Embodiment

Participants described how delays in recognizing Lyme disease manifested physically, emotionally, and socially, illustrating the ecosocial domain of embodiment.

### Delayed diagnosis

Three participants had received a diagnosis via an EM rash; the remainder—twelve participants—were diagnosed after presenting with disseminated forms of Lyme disease, including Lyme arthritis (3), Lyme carditis (3), neurological involvement (3), and flu-like systemic illness (3).

On average, participants required three clinical visits before being diagnosed; two participants required five or more visits. As one participant explained after visiting four different providers:

A physician said, “There is nothing wrong with [you]. You just need to go home and rest.” Assuming I just went home to rest and I rested for a month or three, I don’t know where I would be today. (PT17)^4^.

Across accounts, participants interpreted these delays as stemming from clinicians’ limited awareness of Lyme disease and insufficient engagement with their symptom histories or environmental exposures:

Some of you [may] have a little bit more of a connection with [your HCP]. I can’t really say there was this [type of] relationship with my first doctor. Even though my son was going to him for a while, I can’t really say there was this deep connection or relationship. The second doctor got a gauge of everything, like travel, where we’ve been, what states has he been to, anything that he could find out, to figure out and to track what could be the issue. I feel like the [first] doctor could’ve done a better job at that. (CG01).

[I think my child would have been diagnosed sooner] if in the hospital, they would have asked more questions about not just treating the fact that he had chest pain, but asking about overall how he’s feeling and what’s going on. They could have tested him in the hospital for Lyme disease. […] Sometimes, I wonder if [when] the doctor sees me and they see him, if there’s an unconscious bias to not give the same care that they would give if my son looked like he could be the doctor’s grandson or something. (CG02).

These narratives reflect a perceived lack of clinical curiosity, which is an omission that participants felt shaped their bodies’ progression into more severe disease states, as when CG02 articulated that this lack of clinical curiosity could be rooted in the difference in race between her son and his HCP.

Similarly, PT17 was aware of something wrong with her skin, but felt disempowered when she attempted to draw attention to this while seeking a diagnosis. She described feeling unsupported and made to feel like she was “going crazy,” which allowed her symptoms to progress before receiving a diagnosis:

I think that when I first walked into the center, the doctor said, “How sure are you that it could be Lyme, because I see nothing on your skin. I’m supposed to see something on your skin, but nothing seems to be there.” […] I think it is because of my skin. Some people don’t get to see what’s wrong. […] I think that is a disadvantage [of being a person of color seeking healthcare]. (PT17).

As reflected in the passages above, most participants were diagnosed only after the disease had progressed to more severe, disseminated stages, with relatively few identified early through hallmark signs like the EM rash. The need for multiple clinical visits signals a pattern of missed opportunities, where symptoms were repeatedly evaluated, but not meaningfully synthesized. Participants didn’t see these experiences as neutral lapses. Several participants pointed to a perceived absence of “clinical curiosity” (a failure to fully investigate patients’ concerns) and linked this to social distance and, in some cases, racial bias. Caregivers and patients alike suggest that differences in race may shape how seriously symptoms are taken, how thoroughly histories are explored, and ultimately how quickly diagnoses are made.

Delayed diagnosis is not merely a function of disease complexity, but of how attention, trust, and diagnostic effort are unevenly distributed, shaping both the trajectory of illness and patients’ experiences of care.

### Emotional impacts

Participants described profound emotional impacts that unfolded after their diagnosis, illustrating how prior clinical encounters and the uncertainty of their illness became embedded in their ongoing relationship with the outdoors. Many expressed persistent worry or vigilance when entering natural spaces, including woods, parks, and even their own yards. They reported avoiding activities they previously enjoyed, such as hiking and biking, due to a sense of vulnerability:

I love a green environment, like, with so many trees and grasses, but those are the places that are prone to have different kinds of insects that just jump on you. I try to avoid too much green. (PT15).

Getting Lyme disease [in the woods]…makes me reluctant to go back. I have that fear now. […] I’ve got the heebie-jeebies. I did go back once, but I’m constantly looking on the ground, and I’m checking myself, checking my legs. That’s not fun to me. (PT04).

These accounts suggest that the experience of delayed diagnosis and illness not only affected participants’ health, but also reshaped their sense of safety and belonging in outdoor environments, transforming once restorative spaces into sources of anxiety and hypervigilance.

Participants also described changes in interpersonal dynamics and self-perception. CG02, reflecting on their child, noted, “It has really changed who he is as a person,” because her son is more fearful, shy, and reserved after his experience with Lyme disease:

He’s very fearful of things. He does not go camping. He does not like to be out in the woods. He will not go outside in the grass and walk. It has really changed who he is as a person.

Another patient expressed how he went from being a “fun guy” to someone new:

Before I was diagnosed with Lyme disease, I used to be this happy person. […] I used to be the fun guy that walked into the room, and everybody wanted to hear me talk…. After [contracting Lyme disease], I think a part of me changed a bit. I dropped a bit with being active and being the very fun guy around people. (PT02).

These narratives indicate that Lyme disease extended beyond physical illness to alter participants’ identities and social worlds, as lasting fear and diminished vitality reshaped how they saw themselves and engaged with others.

### Physical impacts

Physical symptoms, such as joint pain, fatigue, weakness, and reduced stamina, continued well after diagnosis, and recovery could take a long time. Participants emphasized how these embodied consequences disrupted daily functioning:

It’s drastic…the way it has affected me negatively. […] Most times, I want to go swimming [but I cannot] because I’m having some kind of pains, some kind of swelling around my joints, and a rash can be itchy and all that. And weakness and all. I feel it’s really, really tiring for me. It’s very challenging. (PT06).

[Contracting Lyme disease] was a huge negative impact on my life. […] [My friends and I] thought we should jog. I noticed I was really, really struggling to breathe…. The second that I was running, it was painful. (PT08).

Participants in recovery expressed ongoing physical impacts, particularly weakness and fatigue, such as PT14, whose daily physical activity was impacted:

Before, I exercised sometimes six times in a week, but now it’s just two or one. […] Because I feel more weak now, […] having the fatigue, the body aches, I won’t be able to go and get things done on my own sometimes. (PT14).

These accounts underscore how the lingering physical burden of Lyme disease constrained participants’ independence and everyday activities, revealing recovery as an uneven and prolonged process marked by ongoing limitation rather than a clear return to prior health.

### Social impacts

Participants described how illness altered their social life. Ongoing physical symptoms influenced social engagement and recreation. Some participants withdrew from outdoor spaces entirely, while others limited travel, gatherings, or leisure activities:

I would say it [has] changed. I reduced my outings. I reduced the fun that I had. I haven’t really taken a lot of trips. It’s my diagnosis. (PT15).

I would say my personal life was off. I was getting more tired and fatigued, so playing video games was my means of relaxation, I would say. I spent more time playing video games. Sometimes, I wouldn't even have the urge to play games. Most of the days I spent on my bed or at home. It affected my social life as well. I spent a lot of time indoors for a few weeks. (PT16).

Participants also reported that fatigue and other symptoms interfered with employment and academic responsibilities:

Because I was getting tired, and it was affecting my work rate. It was affecting my work rate, and sometimes my boss complains that I’m not proactive at work. I didn’t want to lose my job. (PT02).

I missed a good number of classes during those few weeks. Finally, I had to go to class. I finally had to get up doing homework, assignments, and projects I had. It affected my school life in the meantime. (PT16).

Participants’ narratives show that the consequences of delayed diagnosis extend beyond individual symptoms to reorganize their social worlds and daily life. Fatigue and pain did not simply limit activity. They restructured how participants engaged with their lives, narrowing their participation in work, school, and relationships. What emerges is not just reduced engagement, but a form of social contraction, where energy becomes a scarce resource that must be carefully rationed. In this way, the embodied effects of Lyme disease are not confined to the body itself, but ripple outward, constraining mobility, productivity, and connection and reshaping participants’ trajectories.

#### Cumulative exposures

Three participants described frustration with repeated healthcare encounters that failed to yield answers, highlighting how cumulative clinical dismissal eroded their confidence in the healthcare system:

Any other time I have had an issue physically, we go to the doctor, and they tell us what to do. […] [Seeking care for Lyme disease] turned out to not be that simple. What made it difficult was going back to…[physicians that] didn’t really provide any solution. It was just frustrating continuing to go to appointments without any real answers. (CG02).

Several participants described adopting assertive self-advocacy strategies as a form of protection, given their knowledge of racial bias in the healthcare system and prior experiences. For example, PT04 described himself as a patient who comes to appointments “armed with knowledge” so that doctors cannot ignore his inquiries:

I’m the type of person [who] demands treatment. I demand to be treated just like anyone else, and I won’t take a back seat to anything or anyone. That’s just the way I am. I deserve to be treated no differently or just as well as someone else with another complexion. […] I demand it, even with my doctors.

In these examples, we see tension between participants’ agency and external constraint. Some participants were able to assert self-advocacy, and this agency was exercised in response to—and also limited—by an underlying lack of trust and inequitable care. In this way, participants’ efforts to demand appropriate treatment reflect both resilience and the additional burden of navigating a system where equitable attention is not assumed.

Four participants described feeling safer and more supported when they were seen by healthcare providers who were also people of color. For these participants, their trust was situated within a broader context of shared social experience and perceived understanding of racialized vulnerability in healthcare settings. As PT12 said:

I just felt that I liked the fact that I got attended to by a person of color. I felt safer.

And PT16 concurred with:

Our primary care physician is also Black as well… it's more convenient. He understands… it's more comfortable.

Participants thus suggested that shared racial or cultural background fostered both implicit and explicit understanding, reducing the need to justify symptoms or advocate for legitimacy.

Among participants who described positive care experiences, strong long-term relationships with healthcare providers were viewed as protective factors that mitigated diagnostic delay and treatment uncertainty. These relationships were characterized by trust, communication, and a sense of being known as a whole person rather than a clinical case:

I can’t say enough good things about my doctor, which is why I trust him with my life. He gives it to me straight. I know he cares about me. I’m not just a number to him or a patient. My life is literally and figuratively in his hands at times. I trust him totally. (PT04).

PT11 further stated:

I will say the relationship with my healthcare provider is a very close one, that I can always trust them. They are my go-to, so whenever I feel anything different, they are the first person that comes to mind.

These accounts suggest that relationships with racially concordant healthcare providers can serve as critical buffers against uncertainty, fostering trust, deeper communication, and more attentive care that supports timely diagnosis.

Participants who reported poor relationships with healthcare providers during the experience of being diagnosed with Lyme disease described longer diagnostic pathways and greater emotional distress. These repeated experiences were characterized by clinicians’ failure to connect symptoms to Lyme disease and participants’ perception that their concerns were minimized:

I felt like this guy doesn’t know what he’s saying. He probably doesn’t know what he’s doing. I had a doubt at that time. He kept telling me I’m fine, and I know when I’m not fine and want to know what’s up. (PT17).

It just seems like I would go from the cardiologist, who never thought of suggesting Lyme disease. […] He just said, ‘”The heart thing is fine. He’s fine.” I didn’t feel supported because I feel like he should dig a little bit deeper to figure out what the cause of all these ailments were. (CG02).

These same participants explicitly linked these experiences to racial discrimination, describing how skin color shaped diagnostic visibility and clinical attention:

I felt discrimination because I had no knowledge about [Lyme disease]. […] [The doctor] said, ”It’s not apparent on your skin. If you were White, I could see it clearly, and I could know that you had Lyme.” I felt the discrimination. That was why earlier I talked about the skin color, [because] if I was White, it could be caught sooner, it could be caught and treated in time. (PT17).

My son is biracial. He’s half Black and half White. Sometimes, I wonder if the doctor sees me and they see him, if there’s an unconscious bias to not give the same care that they would give if my son looked like he could be the doctor’s grandson or something. (CG02).

These narratives illustrate how repeated exposure to perceived bias accumulated to heighten mistrust, prolong uncertainty, and potentially exacerbate disease progression. Importantly, they also reveal how diagnostic practices themselves can be structured around clinical norms (e.g., the visibility of rashes on lighter skin) that systematically disadvantage people of color. In this way, participants’ experiences point to an interaction between interpersonal bias and structural limitations in medical knowledge, where inequities are reproduced, not only through how patients are treated, but through what clinicians are trained to see.

#### Accountability and agency

Participants varied in their familiarity with Lyme disease before diagnosis. Those with little prior awareness described confusion and fear upon learning of their condition, reflecting gaps in public health education and community-level dissemination of information. PT01 said:

My first thing was, ‘Lyme disease. What is this, and do you keep it? Do you get rid of it?

And PT03 recalled:

I was scared. I was like, “What is this? How did I get this? What disease? Is it sexually transmitted or what?” [The doctor] had to explain all of that to me. He spoke to me at length, and I felt calm. And I just followed the whole process.

The accounts suggested that responsibility for early recognition of Lyme disease was implicitly placed on individuals, despite the absence of accessible or culturally relevant information.

Participants also described being unaware that people of color could contract Lyme disease. This perception was shaped by racialized narratives about outdoor recreation and environmental exposure as well as the absence of visible examples of people of color discussing Lyme disease publicly:

I feel like, for me, it wasn’t something that I thought [about]. […] I know this is going to sound so ignorant, [but] I didn’t even think Black people got Lyme disease. I thought it was, like I said, other cultures may get it that do camping and more hiking and outdoorsy activities. (CG02).

PT01 further reflected:

I don’t think most Black folks are known for going hiking and camping as much as we are to be more urban dwellers. I like to be urban and rural. I am definitely the contradiction to the stereotype.

These accounts suggest that racialized assumptions about who is at risk for Lyme disease can obscure recognition and delay care, as limited representation and dominant narratives about outdoor exposure shape both awareness and perceived susceptibility among people of color.

Participants who were familiar with Lyme disease prior to diagnosis often attributed their knowledge to exposure through predominantly White social networks or public figures. For example, some learned about Lyme disease through White coworkers in outdoor occupations, while others referenced celebrities who had publicly discussed their illness.

As CG01 said:

I think one of the famous models and their mom has Lyme disease.

And PT04 further elaborated:

I knew about [Lyme disease] for years because I work in the construction industry…. In my field, 95% of the people are White. Every hunting season, they’ll all take off. I’ve been around them for almost 40 years, so I know about Lyme disease by proxy.

These memories highlight how access to information is mediated by social proximity and representation.

When reflecting on their experiences, participants consistently emphasized the need for broader, systemic awareness efforts. Rather than framing lack of knowledge as an individual failing, they pointed to hospitals, community spaces, and public health institutions as key sites of responsibility. For instance, PT11 stated that:

If there’s more awareness created on this, people would not just assume it’s flu.

And PT12 opined:

I just think an awareness should happen, probably in hospitals and community centers, so people would know about issues and symptoms related to diseases like this.

Participants further reflected on the importance of early recognition and prevention, emphasizing how greater awareness could have altered their illness trajectories, as when PT04 said:

I wish I had known more… I would have taken some precautions.

Participants framed knowledge about Lyme disease as unevenly distributed across race, wherein awareness is often accessed “by proxy” through proximity to White networks, underscoring a lack of institutional accountability in disseminating inclusive public health information. At the same time, participants assert a form of agency in calling for systemic change, positioning hospitals, community spaces, and public health systems as responsible for closing these gaps so that individuals are better equipped to recognize risk and take preventive action.

## Discussion

This study offers in-depth insight into the experiences of people of color with Lyme disease and contributes to emerging scholarship demonstrating how historically and socially patterned exposures and vulnerabilities shape illness experiences. Participants’ narratives illustrate how social and structural conditions are embodied over time, influencing not only health outcomes, but also pathways to diagnosis, treatment, and recovery. By centering the perspectives of people of color, who are underrepresented in Lyme disease research, yet disproportionately experience more severe disease manifestations, this study makes an original contribution to the health disparities literature and advances understanding of how racialized social structures shape infectious disease experiences in the U.S. Further, it also highlights those experiences that may be universal to people of color seeking healthcare, as well as universal aspects to the Lyme disease patient experience regardless of patient race or ethnicity.

Consistent with ecosocial theory’s emphasis on embodiment, participants’ experiences extended beyond physical symptoms to encompass shifts in identity, family relationships, work and school participation, and engagement with outdoor environments. Participants described anxiety, depression, and persistent worry, particularly related to being outdoors, that altered daily activities and social participation. These emotional responses represent a form of embodied inequality, where delayed diagnosis, fear of reinfection, and the memory of severe symptoms shape one’s feelings, behaviors, and sense of safety. While similar emotional distress has been documented among White people with Lyme disease (Drew & Hewitt, 2006), this study highlights how such impacts intersect with delayed diagnosis and more severe disease manifestations, which are disproportionately experienced by people of color (Gould et al., 2024, Starke et al., 2023).

Across participants’ accounts, prior healthcare encounters, awareness of racialized bias in the healthcare system, and ongoing structural barriers accumulated throughout Lyme disease journeys influence both diagnosis and recovery. Participants described how racialized assumptions about Lyme disease risk and the lack of visible representation of people of color in Lyme-related narratives constrained awareness before diagnosis. Awareness of Lyme disease flowed through racially stratified social networks, mediated by the experiences of White acquaintances or public figures. Participants described limited awareness of Lyme disease prior to diagnosis, suggesting that even in high-incidence settings, knowledge about Lyme disease does not circulate equitably (Shafquat et al., 2025). These findings raise concern that challenges may be even more pronounced for people of color living in emerging risk areas, where general awareness of Lyme disease is lower, and public health infrastructures are still adapting to changing epidemiological patterns.

Participants’ experiences with healthcare providers further illustrate how structural inequities are reproduced in clinical encounters. Participants described delayed diagnoses, difficulty having symptoms taken seriously, and clinicians’ inability to recognize EM on darker skin tones. These experiences were often interpreted through participants’ broader awareness of racial bias in healthcare, shaped by personal histories and collective knowledge of how people of color are treated in medical settings. This awareness accumulated over time and informed how participants approached care, including decisions to seek providers with whom they had established relationships or who shared their racial or ethnic background. Consistent with prior research, these findings support theories that implicit and explicit healthcare biases contribute to racialized disparities in Lyme disease outcomes (Gould, Fathalla, et al., 2024) and echo longstanding concerns about inadequate clinical training on dermatologic variation across skin tones (Dodd et al., 2023, Ebede and Papier, 2006, Nolen, 2020). In response to these gaps, the Skin of Color Society offers references and mentorship to dermatologists to increase knowledge and awareness of the treatment of skin disease with people of color (Skin of Color Society, n.d).

At the same time, participants’ narratives reflect agency alongside constraint. Participants articulated recommendations for improving Lyme disease education, diagnosis, and prevention, emphasizing the need for inclusive and culturally responsive public health messaging. Such findings align with recent efforts by public health agencies, including the Centers for Disease Control and Prevention (2024a), to expand educational materials that reflect diverse skin tones and lived experiences. However, participants’ accounts suggest that meaningful progress will require sustained structural investment and engagement with communities historically excluded from Lyme disease narratives. Beyond general cultural competency training, providers could also engage in continued education specifically about identifying Lyme disease in people of color (e.g., identifying EM rashes on non-White skin tones) and how to provide culturally appropriate resources to people of color in high-incidence areas.

### Limitations and future research

The present study reflects the feelings and experiences of participants who were mostly Black and living in the Northeastern U.S. and thus are not necessarily generalizable to other groups. Though code saturation was achieved with this sample, future studies may consider the experiences of other people of color (non-Black people of color living outside the Northeast), from which new themes could arise. Also, because of the challenges in recruiting this subpopulation of patients with Lyme disease, this study included patients with a range of Lyme disease manifestations, and people with disseminated disease were overrepresented compared with Lyme disease patients overall. It is possible that there are variations in experience depending on disease stage that were not elucidated in this study. Further, challenges among participants in low incidence states may face even more difficulties, due to lack of awareness. Participants were recruited from online groups discussing Lyme disease, and participants recruited in other ways may have different experiences than the ones presented here.

It is possible some case misclassification occurred, as participants were self-identified as having Lyme disease and their diagnosis could not be confirmed via medical records. However, to increase the likelihood that participants included in this research truly experienced Lyme disease, we included several questions in the screener to confirm diagnosis, such as asking for the type of Lyme disease participants were diagnosed with and asking for specific tests.

While patients reported their state of residence, they did not report the state where they received their care. To protect the identities of patients, interview questions did not probe for specific locations for where they received care, and the assumption was made that care was received in the same state where they resided. However, it is possible that patients contracted Lyme disease in a different state or sought care in another area, which may have changed their diagnosis experience.

Finally, there may be variation in experience depending on the stage of disease. This study included all disease stages; therefore, experiences varied from participant to participant.

## Conclusion

Taken together, these findings demonstrate that disparities in Lyme disease among people of color cannot be attributed solely to individual behaviors or knowledge gaps. Rather, they reflect patterned inequities produced through racialized assumptions about risk, uneven dissemination of public health information, and structural biases embedded within healthcare systems. Addressing these inequities will require approaches that move beyond individual vigilance to confront the institutional practices and social narratives that shape who is recognized as being at risk.

## Ethics Approval and Informed Consent

The study protocol was reviewed for ethical consideration and granted exempt status by Pearl IRB, an independent review board, in November 2023. All qualified participants who agreed to participate provided informed consent.

## CRediT authorship contribution statement

**Gabriela Burgos:** Writing – review & editing, Resources, Project administration, Conceptualization. **Stephanie A. Duench:** Writing – review & editing, Supervision, Funding acquisition, Conceptualization. **L. Hannah Gould:** Writing – review & editing, Supervision, Funding acquisition, Conceptualization. **Alexandra Kissling:** Writing – review & editing, Writing – original draft, Validation, Methodology, Formal analysis, Conceptualization. **Kathleen Beusterien:** Writing – review & editing, Writing – original draft, Validation, Methodology, Formal analysis. **Lewis Kopenhafer:** Writing – review & editing, Resources, Project administration, Conceptualization.

## Declaration of Competing Interests

The authors declare the following financial interests/personal relationships which may be considered as potential competing interests: Stephanie A. Duench and Hannah Gould are employed by Pfizer and may hold stock or stock options. Alexandra Kissling, Kathleen Beusterien, Lewis Kopenhafer, and Gabriela Burgos are employed by Oracle Life Sciences, a commercial business unit of Oracle Corporation. These affiliations do not alter the authors' adherence to journal policies on data sharing, transparency, and research integrity.

## Footnotes

4 Participants were assigned a unique identifier at the recruitment screening and enrollment stage to protect their anonymity. Acronyms “PT” and “CG” were used to denote if a participant is a patient or caregiver.
